# An environmental evaluation of urine-diverting dry toilets in Hiloweyn Camp, Dollo Ado, Ethiopia

**DOI:** 10.1016/j.scitotenv.2024.171838

**Published:** 2024-03-20

**Authors:** Travis W. Brown, Jennifer L. Murphy, Patricia Akers, Molly Patrick, Vincent Hill, Mia Mattioli, Yegerem Tsige, Ahmed Adow, Mohamed Abdirashid, Mohamed Nur Mohamed, David Githiri, Thomas Handzel

**Affiliations:** a Emergency Response and Recovery Branch, Division of Global Health Protection, Centers for Disease Control and Prevention, Atlanta, GA, USA; b Waterborne Disease Prevention Branch, Division of Foodborne, Waterborne, and Environmental Diseases, Centers for Disease Control and Prevention, Atlanta, GA, USA; c United Nations High Commissioner for Refugees, Switzerland; d Norwegian Refugee Council, Norway

**Keywords:** Urine-diverting dry toilets, Sanitation, Humanitarian emergencies, Pathogen inactivation, *Ascaris* ova, *E. coli*

## Abstract

Safe and hygienic management of human waste is essential in humanitarian settings. Urine-diverting dry toilets (UDDTs) can enable this management in some humanitarian emergency settings. A seeded, longitudinal environmental study was conducted in Hiloweyn refugee camp, Dollo Ado, Ethiopia, to measure *Escherichia coli* and *Ascaris suum* ova inactivation within closed UDDT vaults and to document environmental conditions (temperature, moisture content, and pH) that could influence inactivation. Hiloweyn camp represented an optimal location for a desiccation-based sanitation technology such as the UDDT. *E. coli* and *Ascaris* ova inactivation was observed in UDDTs under warm, dry, alkaline conditions at 6, 9, and 12 months of storage; UDDTs with samples containing *<*1000 *E. coli*/g total solids increased from 30 % to 95 % over 12 months, and a *>*2.8-log_10_ reduction in *Ascaris* ova viability was observed after 6 months. Additional laboratory-based studies were conducted to provide insights into the field study findings and study the impact of hydrated lime on *E. coli* and *Ascaris* ova inactivation. Results suggest that adding hydrated lime to elevate pH *>* 12 may increase inactivation and decrease storage time. Overall, UDDTs could contribute to the safe and hygienic management of human waste in comparable warm and dry humanitarian settings.

## Graphical Abstract

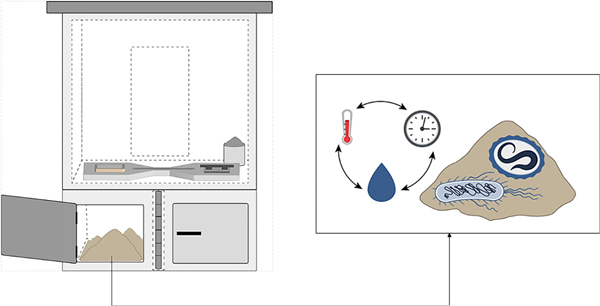

## 1. Introduction

**Fig. 1.**
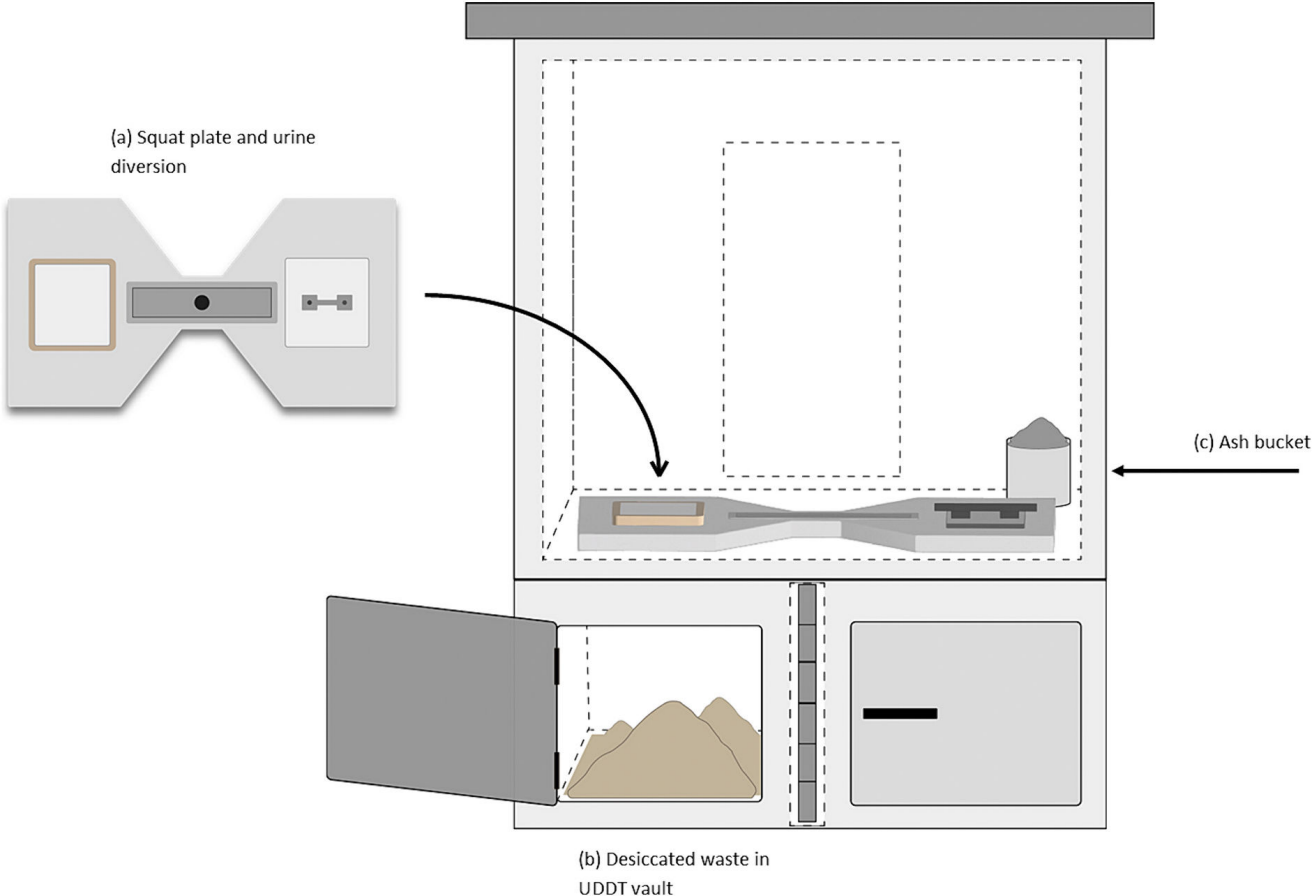
Schematic of UDDT design specifically implemented in Hiloweyn Camp, Dollo Ado, Ethiopia. (a) Squat plate and urine diversion slab; (b) One of two alternating vaults containing desiccated human waste; (c) addition of ash encouraged after each defecation to further promote desiccation.

Urine-diverting dry toilets (UDDTs) are an above-ground dry excreta management system. UDDTs separate urine and feces at the squat plate or pedestal seat; in a double-vault system, feces are collected in one of two alternating collection vaults (Fig. 1). The “active” vault is used to collect feces, and the “closed” vault is used to store previously-collected feces for microbial inactivation, primarily through desiccation, for a period of 6 to 12 months (Rieck et al., 2012). After the storage period, the contents of the closed-vault are manually removed and transferred to a secondary location; the emptied vault then becomes the in-use, active vault. The adjacent, previously active, and now full vault is then closed for a determined storage period (e.g., 12 months), or until the active vault becomes full. As an above-ground sanitation system, UDDTs provide an alternative to traditional sanitation options, such as pit latrines, offering longer lifespan and space-saving infrastructure as they can be emptied and reused (Gensch and Jennings, 2018). Furthermore, UDDTs are an option when difficult soil conditions, rocky terrains, or flooding prevent pit latrine installation or effective use (Ngala et al., 2014).

UDDTs may play an important role in humanitarian contexts as they could enable safe and hygienic management of human waste where these conditions can commonly occur and may be a suitable option in protracted humanitarian settings in which relevant environmental and social conditions are present (Ngala et al., 2014; Munch et al., 2006). Hiloweyn Camp, a refugee camp in the Dollo Ado district in southeastern Ethiopia, began pilot-scale installation of 90 single-family double-vault UDDTs in 2012 because the rocky soil made it difficult and expensive to excavate for pit latrines in certain areas of the camp. Additionally, the hot, dry climate (mean yearly temperature of ~30 °C; mean yearly rainfall of 236 mm) (Mulugeta et al., 2017) of the Somali Region made Hiloweyn Camp an ideal location for the application of a desiccation-based sanitation technology such as the UDDT. Evidence was needed to determine the appropriate storage time of waste inside closed UDDT vaults to ensure the safe handling and disposal of human waste.

Despite documentation of their use in the humanitarian context, there is limited in situ evidence of the performance of UDDTs in terms of microbial inactivation to reduce risk from waste handling during the emptying of vault contents (Bastable and Lamb, 2012). Evaluations of UDDT performance in El Salvador, Panama, Vietnam, and South Africa demonstrated that higher temperatures, lower moisture content, elevated pH, higher ammonia concentrations, and longer storage times contribute to increased microbial inactivation in UDDT waste (Moe and Izurieta, 2003; Carlander and Westrell, 1999; Buckley, 2008; Mehl et al., 2011). In Hiloweyn, user addition of ash after each defecation was encouraged to promote desiccation and thereby increase microbial inactivation. The use of additives (e.g., lime, ash, sawdust, ammonia) to increase microbial inactivation has been piloted both in lab and field studies, but optimization of these additive inputs under field conditions has not been achieved to date (Austin and Cloete, 2008; Magri et al., 2013).

In 2014, the United Nations High Commissioner for Refugees (UNHCR) and the Norwegian Refugee Council (NRC) requested assistance from the United States Centers for Disease Control and Prevention (CDC) to study the performance of UDDTs in Hiloweyn to determine the effectiveness of the UDDTs in inactivating pathogens, the appropriate storage time needed to ensure the safe handling of the treated feces when the vault is emptied, and to inform additional interventions such as the use of additives at the time of vault closure (or at a secondary storage location).

## 2. Materials and methods

From 2015 to 2016, a longitudinal field study was conducted to assess environmental parameters (temperature, moisture content, and pH) and measure inactivation of the corresponding naturally-present *E. coli* and seeded *Ascaris suum* ova in waste piles of closed UDDT vaults. *Ascaris* ova were seeded into UDDT vaults due to the lack of intrinsic ova in UDDT waste piles observed during a preliminary investigation (data not shown). An accompanying study to assess the acceptability and use of UDDTs in the Hiloweyn camp was conducted concurrently and published elsewhere (Patrick et al., 2021). Over the course of 12 months, environmental parameters and *E. coli* and *Ascaris* ova inactivation in UDDT waste were measured beginning at the time of active vault closure. While waste re-use for agricultural purposes was not an aim of the Hiloweyn UDDT program, *Ascaris suum* ova and *E. coli* were selected as representative microbes for parasitic and bacterial pathogens in waste, respectively, based upon World Health Organization (WHO) guideline values for verification monitoring of treated feces for use in agriculture (World Health Organization, 2006). Based on the results of the longitudinal field study, we subsequently conducted two laboratory studies under controlled environmental conditions to (1) better understand *E. coli* and *Ascaris* ova inactivation in waste at shorter storage times than assessed in the field study and (2) simulate secondary treatment at time of vault closure (or at a secondary off-site storage location) by the addition of hydrated lime.

### 2.1. Longitudinal field study

#### 2.1.1. Selection of UDDTs

A total of 20 shared-family UDDTs (used by 2 or more families) were selected for the longitudinal field study. To be included in the study, the UDDTs had to be currently in use, and the active vault had to be at least 50 % full at the time of the initial visit. Norwegian Refugee Council (NRC) managed all water, sanitation, and hygiene (WASH) infrastructure and services and granted permission for inclusion of UDDTs in the study.

#### 2.1.2. Preparation of Ascaris ova and indicator bags

**Fig. 2.**
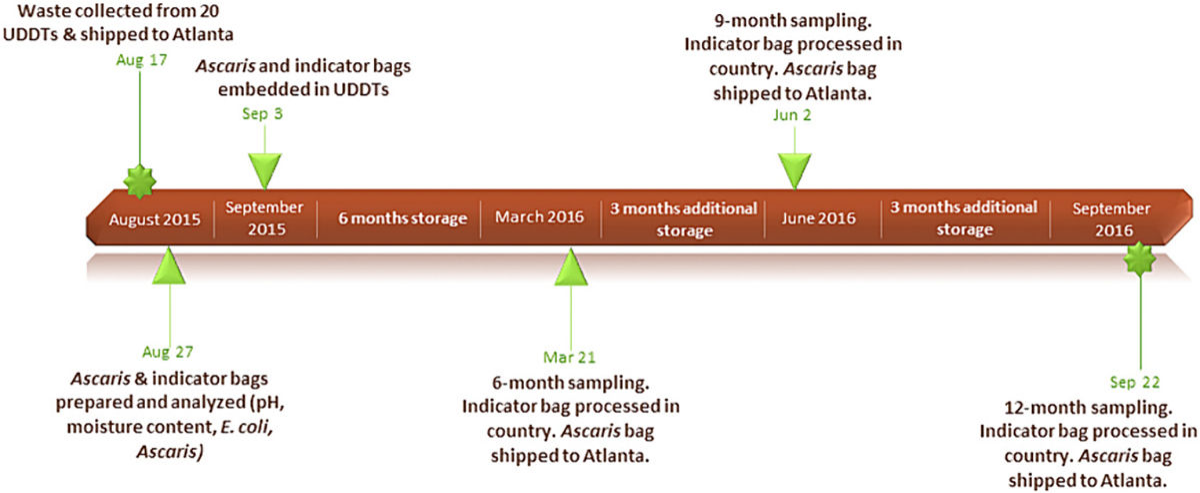
Timeline of events for the longitudinal field study.

*Ascaris* ova were seeded into waste in active UDDT vaults before they were closed, according to the following procedure. Waste from the top and middle layers in the active vault of each of 20 shared-family UDDTs was collected using a hand shovel disinfected with a 3 % high-test hypochlorite (HTH) solution into an individual, new gallon-sized plastic bag (timeline of events is detailed in Fig. 2). A sterile disposable spatula was then used to transfer the material into a sterile 1 l polypropylene bottle. Bottles were shipped on ice packs to CDC laboratories in Atlanta, GA, USA. After waste collection, active vaults were closed and no longer used. For each of the 20 bottles of waste, eight “tea bags” (Jensen et al., 2009) were prepared in the CDC laboratory using sterile 20-micron nylon mesh (Small Parts, Inc; Logansport, IN). Four “*Ascaris* ova bags” each containing 3 g of UDDT waste were seeded with approximately 20,000 *Ascaris suum* ova (Excelsior Sentinel, Inc.; Trumansburg, NY), and bags were sealed with a heat sealer. The 20-micron pore size of the nylon mesh confined the ova within the bag but still allowed them to be exposed to the environmental conditions of the surrounding waste. Four “indicator bags,” each containing 60 g of unaltered and unseeded UDDT waste, were prepared for measurement of moisture content, pH, and autochthonous *E. coli*. One of each bag type was immediately processed to determine baseline conditions using the methods described below. Strings with labels indicating “*Ascaris* ova bag” or “indicator bag” and storage time (6, 9, or 12 months) were attached to the remaining bags. The bags were returned to respective bottles of waste and shipped on ice packs to Hiloweyn Camp.

#### 2.1.3. Seeding bags into UDDTs

The *Ascaris* ova and indicator bags were seeded in Hiloweyn UDDT waste piles 7 days after preparation in Atlanta laboratories. At each of the 20 study UDDTs in the Hiloweyn Camp, a hand shovel was disinfected using a 3 % HTH solution followed by an ethanol rinse. The shovel was then used to carefully remove approximately one-half of the waste pile inside the closed UDDT vault onto an unused polyethylene plastic bag. Three *Ascaris* ova and three indicator bags prepared using material from that same UDDT were then positioned in the center (horizontally) of the waste pile (see Fig. S1 in SI). The hand shovel was used to return removed contents onto the waste pile, covering the seeded “tea bags” completely but allowing strings with labels to remain uncovered for future removal of bags. Additionally, a temperature logger (HOBO; Bourne, MA, USA) was inserted in the top, middle, and bottom layers of two of the UDDTs; the logger recorded temperature every 5 min for the first 6 months of the study, at which point the instruments were non-functional, likely damaged by rodent intrusion inside vaults.

#### 2.1.4. Removal of bags from UDDTs

At 6, 9, and 12 months, one *Ascaris* ova bag and one indicator bag from each of the 20 study UDDTs were removed by gently pulling the labeled bag string. The two bags were placed in a 1 l bottle and additional waste from the UDDT was added to surround the bags and minimize further desiccation (see Fig. S2A in SI). Bottles were first shipped on ice packs to the Ethiopian Public Health Institute (EPHI) in Addis Ababa, Ethiopia, where indicator bags were removed and analyzed for moisture content, pH, and *E. coli* concentration (see Methods below); *Ascaris* ova bags remained in the bottles, surrounded by waste, and were shipped on ice packs to CDC laboratories in Atlanta for *Ascaris* ova enumeration and viability testing. At the time of sampling, the temperature of waste roughly at the top, middle, and bottom regions of the waste pile within each UDDT was measured using a temperature probe (Onset; Bourne, MA, USA).

#### 2.1.5. Analysis of indicator bags

##### 2.1.5.1. Moisture content and pH.

Sterile forceps were used to remove the indicator bag from each bottle and sterile scissors were used to cut bags open. EPA Method 1684 was used to determine the percent total solids (dry weight basis) in samples (United States Environmental Protection Agency, 2001). The percent total solids was calculated as the dry weight divided by the wet weight multiplied by 100. Percent moisture content was calculated by subtracting the percent total solids from 100%. EPA Method 9045D was used to determine pH of samples using a three-point calibrated pH probe (ThermoFisher; Beverly, MA, USA) (United States Environmental Protection Agency, 2004).

##### 2.1.5.2. E. coli quantification.

The autochthonous *E. coli* concentration in each indicator bag was determined by performing a dissolution/elution procedure followed by analysis using a standard most probable number (MPN) assay (Rodger and Bridgewater, 2017). A sterile disposable spatula was used to transfer 25 g of waste from inside the indicator bag into a 500 ml polypropylene bottle containing a magnetic stir bar. Next, 200 ml of sterile elution buffer (0.1 % peptone water with 0.05 % Tween 80) were added to the bottle and contents were stirred on a magnetic stir plate at 250 rpm for 1 h. Solids in the suspension were then allowed to settle for 5 min and a ten-fold dilution series of the supernatant was prepared in sterile dissolution/elution buffer. Ten-ml volumes of select dilutions were added to 90-ml volumes of sterile distilled water and assayed for *E. coli* using the Colilert^®^−18 Quanti-Tray^®^/2000 (IDEXX, Westbrook, ME, USA) method per manufacturer’s instructions (9223 Enzyme Substrate Coliform Test, 2018). Back-calculation was used to estimate *E. coli* MPN per g total solids (dry weight basis) for each sample.

##### 2.1.5.3. Ascaris ova viability.

*Ascaris* ova bags shipped to Atlanta were processed within 7–14 days of collection in Hiloweyn Camp and kept at 4 °C until processing. *Ascaris* ova viability was determined using a filtration and flotation procedure followed by 28-day incubation in 0.5 % formalin and microscopy, as previously described (detailed methods in SI) (Roepstorff and Nansen, 1998; Vadlejch et al., 2011). After incubation, a subset (0.25–0.50 ml) of the incubated solution was transferred to a Sedgewick Rafter counting slide (Graticules Optics; Tonbridge, United Kingdom) and examined under 10× magnification to enumerate viable (i.e., containing larvae) and nonviable ova. *Ascaris* ova were considered viable if fully developed larvae were observed within ova (non-motile or motile) (Ravindran et al., 2019). Counts were used to calculate viable *Ascaris* ova per gram of waste and log_10_ reduction values of ova (viable and nonviable) over time. Viable *Ascaris* ova log_10_ reductions are expressed as log_10_ (N_t_/N_0_), where N_0_ is the number of viable Ascaris ova at baseline and N_*t*_ is the number of viable Ascaris ova at time *t*. If no viable ova were detected, an ovum count of 1 was used for calculations to reflect a max log reduction ranging from *>*2.7 to *>*2.8 log10.

### 2.2. Laboratory studies

#### 2.2.1. E. coli and Ascaris ova survival evaluation

An *E. coli* and *Ascaris* ova survival evaluation was conducted in the laboratory to assess the survival of *Ascaris* ova and *E. coli* in UDDT waste under simulated environmental conditions similar to those observed in Hiloweyn. The purpose of this evaluation was to better characterize inactivation at shorter storage times than observed in the longitudinal field study. Additionally, biosolids procured in Atlanta, GA (“US biosolids”) were included to compare inactivation in fecal waste characteristics that may be found in more humid environments than Hiloweyn (e.g., higher moisture content).

The samples of waste used to surround *Ascaris* ova and indicator bags from the 20 UDDTs of the longitudinal field study (“UDDT waste”) were shipped on ice packs to CDC laboratories in Atlanta and stored at 4 °C for approximately 8 months. In order to mimic moisture content found in the longitudinal field study, a total of 3 kg of well mixed UDDT waste from the field study (collected at the 6 month sampling) was moistened with sterile DI water to the highest moisture content observed (20 %) in UDDT waste at baseline in the longitudinal field study; moisture content was confirmed using EPA Method 1684 as described above. A total of 1 kg of waste was then transferred into each of three sterile 1-l polypropylene bottles.

In March 2018, biosolids were collected from an Atlanta-area wastewater treatment plant (“U.S. biosolids”). The material was dried in an oven at 103–105 °C to achieve a moisture content of approximately 60 % to mimic moisture content that may be observed in more humid tropical environments (Moe and Izurieta, 2003); moisture content was confirmed using EPA Method 1684 as described above. The pH of the U. S. biosolids was not altered to match that of the UDDT waste to avoid the addition of chemical additives that could influence *E. coli* and *Ascaris* ova inactivation. A total of 1 kg of waste was then transferred into each of three sterile 1 l polypropylene bottles.

Ten *Ascaris* ova bags, each containing 20,000 *A. suum* ova (Excelsior Sentinel, Inc.; Trumansburg, NY) seeded directly into nylon “tea bags” without waste (to decrease laboratory processing time), were layered into each of the six bottles; each bag was completely surrounded by waste. Due to difficulties in evenly distributing *E. coli* in 1 kg of waste within 1-l bottles, seven 1 g aliquots of waste from each of the 6 bottles were transferred into 2-ml sterile microcentrifuge tubes containing 10^8^ *E. coli* (ATCC 11775) (ATCC; Manassas, VA, USA) colony forming units (CFU) and mixed by vortex for 30 s.

Immediately after bottles and tubes were prepared, Time 0 conditions were characterized. Moisture content and pH of waste inside bottles was measured as described above. While ammonia measurements were not included in the field study, measurements were included in the laboratory-based studies; uncharged ammonia (NH_3_) is known to have a biocidal effect against many microorganisms, including *Ascaris* ova, in alkaline environments (Pecson et al., 2007; Pecson and Nelson, 2005; Fidjeland et al., 2015). Total ammonia concentration was measured by first preparing waste samples according to the preparation steps of the indophenol blue method for soil samples (Merck; Whitehouse Station, NJ). (. Briefly, a 50-g sample of waste was added to 100 ml of a 0.025 M CaCl_2_ solution with a spatula-tip-full of activated charcoal and mechanically shaken vigorously for 1 h. Total ammonia in the filtered suspension was measured using a Hach test kit (Method 10031), according to the manufacturer’s instructions. One microcentrifuge tube and one *Ascaris* ova bag from each of the six bottles were assayed for *E. coli* concentration and *Ascaris* ova viability, as described above. All bottles and microcentrifuge tubes were then sealed and placed in an incubator at 34 °C.

#### 2.2.2. Hydrated lime treatment evaluation

A hydrated lime treatment evaluation was conducted in the laboratory to assess the survival of *Ascaris* ova and *E. coli* in UDDT waste amended with hydrated lime under simulated environmental conditions similar to those observed in Hiloweyn, simulating secondary treatment at time of vault closure (or at a secondary off-site storage location). A total of 9 kg of well mixed UDDT waste from all 20 UDDTs from the 6-month sampling was moistened with sterile DI water to 20 % moisture content; moisture content was confirmed using EPA Method 1684 as described above. Next, waste mixed with commercially available hydrated lime (Bonide; Oriskany, NY) and aliquoted into nine sterile 1 l polypropylene bottles; each bottle contained a final weight of 1 kg of waste with concentrations of 0.5 %, 2 %, and 5 % hydrated lime (w/w) (*n* =3 for each concentration). As described above, ten *Ascaris* bags were prepared and layered into each of the nine bottles and seven 1-g aliquots of waste from each of the nine bottles were transferred into 2-ml sterile microcentrifuge tubes containing 10^8^ CFU *E. coli*. Immediately after bottles and tubes were prepared, Time 0 conditions (moisture content, pH, ammonia concentration, *E. coli* concentration, and *Ascaris* ova viability) were measured as described above. All bottles and microcentrifuge tubes were then capped and placed in an incubator at 34 °C.

#### 2.2.3. Analysis of waste for laboratory studies

Samples from both evaluations were analyzed at 7, 14, 21, 28, 42, 56, and 84 days. At each time point, moisture content and pH of waste surrounding *Ascaris* ova bags were measured as described above. *E. coli* concentration was also measured in waste at each time point from three UDDT waste tubes and three U.S. biosolid tubes; *E. coli* concentration was only measured in tubes containing UDDT waste plus hydrated lime at 84 days due to large *E. coli* reductions observed at Time 0 and logistical constraints in the laboratory. To measure *E. coli* in the waste, a 1 g sample of waste was added to 8 ml sterile elution buffer and shaken by hand for 5 min. Solids in the suspension were allowed to settle for at least 5 min. The supernatant was serially ten-fold diluted in sterile phosphate-buffered saline (PBS) then analyzed via membrane filtration (United States Environmental Protection Agency, 2002). A positive *E. coli* control (ATCC strain 11775) and a lab blank (10 ml PBS) were analyzed following analysis of all samples at each time point. Back-calculation was used to determine *E. coli* CFU/g total solids for each sample. For samples with no detectable *E. coli*, a colony count of 1 CFU was used for calculations to determine lower detection limit (*<*0.7 and *<* 1.3 log_10_ CFU/g total solids for UDDT waste and US biosolids, respectively).

To enumerate *Ascaris* ova at each time point, one bag was removed from each bottle and washed with sterile deionized water to remove residual waste on the outside of the bag. The bag was aseptically cut open, and the entire bag was transferred into a glass Petri dish containing 10 ml incubation solution (0.5 % formalin). An additional 10 ml of incubation solution was added, directing the stream to elute ova from the bag surface; *Ascaris* bags were not removed from Petri dishes, and dishes were loosely covered with paraffin film. Samples were incubated at 28 °C for a minimum of 28 days with gentle swirling every 2–3 days. A 0.75 ml aliquot of the incubated solution was then transferred to a Sedgewick Rafter counting slide and examined under 10× magnification to enumerate viable (i.e., containing non-motile or motile larvae) and nonviable ova. Counts were used to calculate log_10_ reduction values. Log_10_ reductions in *Ascaris* ova viability at each sampling point were calculated as described above. Slight evaporation of formalin during incubation resulted in final volumes ranging from 15 to 20 ml; because 0.75 ml aliquots were always analyzed, detection limits varied between samples. The upper detection limit for log_10_ reduction values were capped at 2.5 log_10_ as a means to manage left-censored data; this capped detection limit reflects the largest volume observed after incubation (20 ml) and the lowest possible viable ovum count (1 ova).

### 2.3. Data analysis

In the longitudinal field study, WHO guideline values for verification monitoring of treated feces for use in agriculture (*<*1 viable helminth ovum and *<*1000 *E. coli* per gram total solids) were used as a conservative measure of UDDT performance (World Health Organization, 2006; United States Environmental Protection Agency, 2002). However, the lower detection limit of our ova recovery method was unable to meet the *<*1 viable helminth ovum metric. Therefore, we also report log_10_ reductions in *Ascaris* ova viability.

In the laboratory studies, *Ascaris* ova inactivation rates could not be determined due to limitations of low sample size. Instead UDDT waste lime treatments and U.S. biosolids were compared with UDDT control waste using mean log reduction values (LRVs) at the first observed storage time in which a 2-log reduction (T_99_) in viable *Ascaris* ova was measured, and statistical significance was determined using a paired *t*-test with α _=_ 0.05. All statistical analyses were conducted using SAS version 9.4 (Cary, NC).

## 3. Results

### 3.1. Longitudinal field study

At baseline (September 2015), the mean temperatures of waste in the top, middle, and bottom regions within the 20 UDDTs were 32 °C, 33 °C, and 32 °C, respectively (Table 1). The mean percent moisture content was 9 % (range: 3 %–20 %) and the mean pH was 9.0 (range: 8.0–10.6). Waste samples from 30 % (6/20) of UDDTs met the WHO *E. coli* guideline of *<*1000 *E. coli*/g total solids. Geometric mean concentration of *E. coli* in UDDTs not meeting the guideline (*n* = 14) was 6.3 × 10^7^ MPN/g total solids (range: 5.0 × 10^3^–1.2 × 10^8^) (Table S1). Baseline *Ascaris* ova viability was 5133 viable *Ascaris* ova/g total solids of waste (Table 1). Mean percent recovery of seeded *Ascaris* ova from UDDT waste at baseline was determined to be 52 % (data not shown).

At 6 months of storage (March 2016), the mean temperature of waste in the top, middle, and bottom regions in the vaults was 36 °C (Table 1). Temperature logger data indicated that the mean diurnal variation within a 24 h period over 6 months was 1.6 °C (range: 1.1–2.1 °C) (data not shown). The mean percent moisture content of waste decreased to 3 % (range: 1 %–10 %), while the mean pH remained constant at 9.1 (range: 7.3–10.9, Table 1). Waste samples from 74 % (14/19) of UDDTs met the WHO *E. coli* guideline. Geometric mean concentration of *E. coli* in UDDTs not meeting the WHO *E. coli* guideline (*n* = 5) was 1.6 × 10^4^ MPN/g total solids (range: 4.0 ×10^3^–9.1 ×10^4^). Viable *Ascaris* ova were not detected (*<*8 viable ova/g total solids) in the volume of waste analyzed for any of the 20 UDDTs, indicating a *>*2.8 log_10_ reduction from viability at baseline.

At 9 months of storage (June 2016), the mean temperature of waste in the top, middle, and bottom regions in the vaults was 34 °C and the mean percent moisture content of waste was 4 % (range: 2 %–13 %), while the mean pH was 9.1 (range: 7.4–10.4, Table 1). Waste samples from 89 % (16/18) of UDDTs met the WHO *E. coli* guideline. Geometric mean concentration of *E. coli* in UDDTs not meeting the WHO *E. coli* guideline (*n* = 2) was 6.1 × 10^3^ MPN/g total solids (range: 2.9 × 10^3^–1.3 × 10^4^, Table S1). Viable *Ascaris* ova were not detected (*<*16 viable ova/g total solids) in the volume of waste analyzed for any of the 20 UDDTs, indicating a *>*2.7 log_10_ reduction from viability at baseline (Table 1).

At 12 months of storage (September 2016), samples were delayed in transit from Hiloweyn camp to the laboratory in Addis Ababa and not processed until 4 days after collection. Samples were kept on ice packs until processing. The mean temperature of waste in the top, middle, and bottom regions was 32 °C (Table 1). The mean percent moisture content of waste was 3 % (range: 2 %–11 %), while the mean pH was 9.1 (range: 7.8–10.7). Waste samples from 95 % (19/20) of UDDTs met the WHO *E. coli* guideline. The *E. coli* concentration in this single sample was *>*2.5 × 10^5^ MPN/g total solids (Table S1). Viable *Ascaris* ova were not detected (*<*8 viable ova/g total solids) in the volume of waste analyzed for any of the 20 UDDTs, indicating a *>*2.8 log_10_ reduction from viability at baseline (Table 1).

### 3.2. Controlled laboratory studies

#### 3.2.1. E. coli and Ascaris ova survival evaluation

Over 84 days of storage, the mean pH of the UDDT waste and U.S. biosolids ranged from 8.3 to 8.8 and 5.1 to 5.3, respectively, and moisture content remained stable for both UDDT waste (mean = 20 %; range: 19 %–21 %) and U.S. biosolids (mean =62 %; range: 60 %–66 %), as intended (Table 2). Mean total ammonia concentration was 100 mg/l in UDDT waste samples and 683 mg/l in U.S. biosolid samples.

Mean concentration of *E. coli* in the UDDT control waste was 7.9 log_10_ CFU/g total solids at baseline (Table 3). The WHO *E. coli* guideline of *<*1000 *E. coli*/g total solids (*>*3 log_10_ reduction) was achieved in all replicates by day 56. The mean concentration of *E. coli* in the U.S. biosolids was 8.4 log_10_ CFU/g total solids at baseline. The U.S. biosolids met the *E. coli* lower detection limit (*<*1.3 log_10_ CFU/g total solids) by 7 days of storage and continued to remain below the detection limit throughout the 84-day study period, therefore meeting WHO *E. coli* guidelines. A 2-log_10_ reduction in viable *Ascaris* ova was achieved by day 28 and day 84 for the UDDT waste and U.S. biosolids, respectively (Table 4). The LRV of US biosolids at 28 days (0.1-log_10_ reduction) was significantly lower than the UDDT control waste LRV at 28 days (2.4-log_10_ reduction) (*p* = 0.01).

#### 3.2.2. Hydrated lime treatment evaluation

The mean pH of 0.5 % lime treatment UDDT waste was initially elevated (*>*12) at baseline, but pH values decreased to pH 8–9 by 7 days (Table 2). The mean pH of 2 % lime-treated samples remained elevated (*>*12) for 28 days of storage time, then decreased to pH 8–9 by 56 days. The mean pH of 5 % lime-treated samples remained elevated (*>*pH 12) for the duration of the 84-day study period and ranged from 12.5 to 12.8. Moisture content remained stable (mean = 19 %; range: 18–20 %) for the 84-day study period for all 3 conditions, as intended.

Immediately after seeding ~10^8^ CFU *E. coli* into waste (*t* = 0 days), 0.5 %, 2 %, and 5 % lime-treated samples had an *E. coli* concentration of 1.2, *<*0.7, and *<* 0.7 log_10_ CFU/g total solids, respectively, thus all treatments met the WHO *E. coli* guideline of *<*1000 *E. coli*/g total solids (Table 3). After 84 days of storage, all UDDT waste lime-treated samples continued to meet this guideline with concentrations of 0.9, *<*0.7, and *<*0.7 log_10_ CFU/g total solids in the 0.5 %, 2 %, and 5 % lime-treated samples, respectively. For the 0.5 %, 2 %, and 5 % lime-treated samples, a 2-log_10_ reduction in viable *Ascaris* ova was observed at 28, 7, and 7 days, respectively (Table 4). The LRV of the 0.5 % lime-treated waste at 28 days (2.5-log_10_ reduction) was not significantly different than the UDDT control waste LRV at 28 days (2.4-log_10_ reduction). The LRV of the 2 % and 5 % lime-treated waste at 7 days (2.4- and 2.5-log_10_ reduction) was significantly higher than the UDDT control waste LRV at 7 days (0.4-log_10_ reduction).

## 4. Discussion

A seeded, longitudinal field study conducted over a 12-month time period in Hiloweyn Camp, Ethiopia provided a unique opportunity to evaluate the performance of UDDTs in a hot, dry environment, representing an ideal location for a sanitation technology that relies on these conditions for waste treatment. Environmental measures over the field study period indicated UDDT vaults were consistently warm, very dry, and moderately alkaline. The temperature and mean moisture content of waste sampled ranged from 32 °C–36 °C and 3 % to 9 %, respectively, over the 12 month storage period. By comparison, a study in Panama reported average temperature of UDDT waste at 29 °C and a moisture content ranging from 29 % to 67 % after 6 to 10 months of closed storage (Mehl et al., 2011). The low moisture content was likely due to hot and arid conditions and the amount of time the active vaults were in use prior to seeding and closure (~1.5 years). The pH of stored waste was unexpectedly high (*>*9), considering the pH of fresh human feces is 6.6 to 7.0 (Rose et al., 2015). The elevated pH was likely due to user addition of ash after each defecation (approximately 1 cup). While ash is primarily used to further promote desiccation, it can also moderately elevate pH of fecal waste (Hijikata et al., 2016).

Overall, UDDTs in Hiloweyn camp seem effective at reducing viable *Ascaris* ova and *E. coli* concentrations. After 6 months of storage, viable *Ascaris* ova concentrations in waste achieved a *>*2.8 log_10_ reduction in viability compared with baseline. At baseline, 30 % of UDDTs already met the WHO guideline for *E. coli*, likely due to amount of time active vaults were in use prior to closure (~1.5 years); 95 % of UDDTs met this guideline by 12 months. Interestingly, the one UDDT above the guideline was sampled from outside of the indicator bag (see limitations below). While it is possible that *E. coli* were able to survive in small pockets of moisture within the waste, a more likely scenario is that because vaults were not completely sealed, *E. coli* were reintroduced into vaults during storage. Evidence of rodent intrusion was present by destruction of temperature loggers inside closed vaults, and rodents may have introduced fecal matter into the vaults. Based on the microbiological results, a conservative timeframe for storing UDDT waste in Hiloweyn is one-year post-vault closure. Our field study found somewhat similar results to those in the literature despite considerable differences in moisture and pH observed in Hiloweyn Camp. In a seeded, longitudinal field study of UDDTs in Vietnam, low moisture content (21 %–45 %) was determined to have a beneficial effect on helminth ova inactivation; *Ascaris* ova seeded into UDDTs underwent 95 % inactivation after 9 weeks of storage (Carlander and Westrell, 1999). Another performance evaluation of UDDTs in El Salvador found high temperature and pH to be the most important factors influencing inactivation; intrinsic viable *Ascaris* ova and fecal coliforms *>*1000 MPN/g total solids were detected in 59 % and 81 % of UDDTs, respectively, with a mean storage time of 306 days, temperature ranging from 22 °C–36 °C, pH ranging from 6.2 to 13.0, and moisture content ranging from 15 % to 96% (Moe and Izurieta, 2003).

The longitudinal field study was subject to several limitations. First, methodological constraints with our *Ascaris* ova assay restrict our interpretation of the data. UDDTs meeting the WHO guideline value of *<*1 viable *Ascaris* ova per gram of waste could not be determined because the method detection limit was near this value. Second, the study was subject to challenges in the field that may have influenced results. Samples could not be processed for *Ascaris* viability in Ethiopia, so samples were shipped to Atlanta. Decays in viable ova may have occurred; however, cold-storage was maintained during transport and *Ascaris* ova are known to be stable at temperatures *<*20 °C (Naidoo and Foutch, 2017). Also, a delay in shipping from Hiloweyn Camp to the laboratory in Addis Ababa resulted in delayed processing of *E. coli* for the 12-month time point by 4 days. Third, *E. coli* measurements were often taken from waste outside the indicator bag if bags could not be found or samples had to be re-assayed. In this case, the surrounding waste within the bottles in which bags were embedded for shipment were used. Notably, during the 6-month time point inconsistencies in electricity availability resulted in insufficient incubation times for samples, so *E. coli* testing had to be repeated the following day. Fourth, sample bottles with missing indicator bags were handled inconsistently. For example, at the 6 and 9 month samplings, if an indicator bag was not present in the bottle, *E. coli* concentrations using waste outside the indicator bag were not measured. However, at the 12 month sampling, outside waste was used. Interestingly, this UDDT sample at 12 months measured above the WHO guideline value for *E. coli*. As vaults were not completely sealed, recontamination of UDDT waste may have been measured when sampling from outside the indicator bag.

In the *E. coli* and *Ascaris* ova survival evaluation, a *>*2-log_10_ reduction in viable *Ascaris* ova in untreated UDDT waste was achieved by day 28, and no viable ova were detected after 56 days. Comparatively, the U. S. biosolids saw a *>*2-log_10_ reduction by day 84, suggesting UDDT waste was a more inhospitable environment for *Ascaris* survival. These results support our findings in the field study that conditions in Hiloweyn camp and subsequent UDDT waste characteristics were unfavorable to *Ascaris* survival. While studies evaluating *Ascaris* ova inactivation in dry fecal matter (≤20 % moisture content) are limited, a recent study evaluating inactivation in soil at a similar moisture content and temperature (*<*20 % and 35 °C) observed a 3 log_10_ reduction after 57 days of storage (Senecal et al., 2020). *E. coli* concentration in all samples met the WHO *E. coli* guideline after 56 days. In the field study, this was never achieved. However, as noted above, recontamination of UDDT waste may have been measured when sampling from outside the indicator bag.

The laboratory-based hydrated lime treatment evaluation demonstrated that the addition of 2–5 % hydrated lime to elevate pH *>* 12 can assist in *E. coli* and *Ascaris* ova inactivation and potentially shorten storage times. In UDDT waste amended with 2 % and 5 % (w/w) hydrated lime, *>*2-log_10_ reductions in *Ascaris* ova viability were achieved at the first sampling time point (7 days). All hydrated lime treated samples met the WHO *E. coli* guideline immediately after treatment. The data suggests that rate of inactivation increased with the addition of lime at 2 % and 5 % when comparing treatment and control LRVs at their T_99_ values. Efficient alkaline treatment is dependent upon concentrations of ammonia in waste, as the elevated pH converts charged ammonia to its biocidal uncharged form. Urine separation from waste may decrease ammonia concentrations in UDDT waste; however, results suggest that concentrations were sufficient to aid in *E. coli* and *Ascaris* ova inactivation. Despite differences in waste characteristics, a study evaluating the effects of temperature, pH, and ammonia on inactivation of *Ascaris* ova showed similar results with a calculated T_99_ of 24 and 3.4 days in sludge (~94 % moisture content) amended with hydrated lime (concentration not reported; pH *>* 12) at 30 °C and 40 °C, respectively (Pecson et al., 2007). Together, our results highlight the importance of monitoring relevant factors when assessing the effectiveness of waste treatment. However, the hydrated lime results should be adopted to field conditions with caution as higher percentages of hydrated lime may be necessary to maintain elevated pH of waste in UDDT vaults that are exposed to the outside environment.

The laboratory studies were subject to several limitations. Methodological constrains with our *Ascaris* ova assay prevented us from seeing large reductions in viability, mainly due to a small percentage of sample being analyzed by microscopy (~3–5 % of total sample volume). Additionally, it was difficult to compare LRVs across lime treatments since percentage of sample analyzed varied. Another limitation was that the statistical analysis of data from the controlled laboratory experiments was limited due to the small number of samples with detectable ova; rapid inactivation of 2 % and 5 % lime-treated samples was unexpected. Furthermore, day 42 results were excluded due to error in sample measurement. And finally, it is difficult to compare results of the *E. coli* and *Ascaris* ova survival evaluation conducted in the laboratory to results from the longitudinal field study due to inability to exactly simulate the observed field conditions. Although temperature, pH, and moisture content were controlled, the microcosm conditions were inherently different due to smaller quantities of waste and tightly closed containers.

In the field study, WHO guideline values for verification monitoring in treated feces for use in agriculture were used as a conservative measure of UDDT performance. The number of UDDTs meeting this metric could not be determined, because the method detection limit was restricted to *<*6 and *<*8 ova/g total solids. However, rough estimates can be calculated to determine if the measured LRVs are enough to achieve this guideline given a hypothetical *Ascaris* carriage prevalence in the community utilizing the UDDT. A study in Bangladesh reported the mean *Ascaris* ova concentration in the stools of infected children to be 1778 ova/g (Hall and Nahar, 1994). Assuming there are 10 users per UDDT, an infection rate of 13 % (observed in a 2016–2018 parasitological survey conducted in Nigeria), and daily fecal load of 150 g/person/day, the *Ascaris* ova concentration would be 178 ova/g in a well-mixed UDDT (Rose et al., 2015; Mogaji et al., 2020). A 2.8-log_10_ reduction (observed in our longitudinal field study after 6 months) of this estimated concentration would meet this guideline at 0.5 viable ova/g total solids. Furthermore, if vaults needed to be closed before recommended storage time and 5 % (w/w) hydrated lime was added to the waste and well mixed, a resulting 2.5-log_10_ reduction after 7 days of storage would meet this guideline at 0.7 viable ova/g total solids. It is important to note that UDDTs may not be well mixed in practice, and there may be small pockets within the waste pile with higher concentrations of ova or where ova may find suitable conditions to survive. For this reason, a factor of safety may be applied to recommend a storage time of 1-year post-vault closure (without hydrated lime treatment). Proper use and maintenance of UDDTs, including regular mixing of waste is important to maximize treatment.

## 5. Conclusions

The hot, dry conditions in Hiloweyn Camp provided an opportunity to assess the performance of UDDTs in a unique environment. This study provides an evidence base for the ability of UDDTs to provide effective management of human waste in comparable humanitarian settings where traditional sanitation options are not feasible and where waste reuse is not an aim of the program. To further assist with UDDT guidance and determination of appropriate settings for use, additional research in more temperate and humid environments is needed. Additional research is also needed to understand the benefit of promoting the behaviors which further desiccate waste (e.g., addition of ash) and performing secondary treatment (e.g., addition of lime), as these may improve UDDT performance in a range of settings. Overall, UDDTs appear to be an effective option in settings with the ability to install permanent infrastructure, with sufficient land space for secondary waste storage and/or treatment and disposal, and with strong oversight to ensure that they are safely used and managed. Effective treatment of UDDT waste could mean an additional resource for land-management and biosolid amendments for food production, but further work is needed to explore this, particularly in refugee settings.

## Supplementary Material

Supplementary Info_UDDT

## Figures and Tables

**Table 1:** Physical/chemical parameters and E. coli and Ascaris suum ova reductions in 20 shared-family UDDTs in Hiloweyn Camp at baseline (0 months; September 2015) and after 6, 9 and 12 months of storage.

Storage Time	Temperature (°C)^a^			Moisture content (%)^a^	pH	No. (%) UDDTs with *<*1000 *E. coli*/g total solids	No. viable *Ascaris* ova/g (LRV^b^ from baseline)
	Top	Middle	Bottom				

Baseline	32	33	32	9 (3–20)	9.0	6/20 (30 %)	5133
	(30–34)	(30–34)	(26–34)		(8.0–10.6)		
6 months	36	36	36	3 (1–10)	9.1	14/19 (74 %)^c^	<8 (>2.8)
	(32–41)	(33–38)	(33–39)		(7.3–10.9)		
9 months	34	34	35	4 (2–13)	9.1	16/18 (89 %)^d^	<16 (>2.7)
	(32–35)	(33–37)	(33–37)		(7.4–10.4)		
12 months	32	32	32	3 (2–11)	9.1	19/20 (95 %)^e^	<8 (>2.8)
	(29–33)	(30–34)	(30–34)		(7.8–10.7)		

a Physical/chemical parameters presented as mean (min–max).

b LRV = log reduction value.

c *E. coli* data for one UDDT are not available because the indicator bag could not be located in the waste pile. Due to inconsistent electricity, *E. coli* measurements repeated the following day using waste from inside bottles in which bags were embedded for shipment.

d *E. coli* data for two UDDTs are not available because the indicator bags could not be located in the waste piles.

e The 12 month indicator bag for one UDDT could not be located in the waste pile. For this UDDT, *E. coli* analysis was performed using the waste material in which its *Ascaris* bag had been embedded for shipment.

**Table 2:** Mean pH and moisture content in untreated and lime-treated (0.5 %, 2 % and 5 % (w/w)) UDDT waste and US biosolids at 34 °C for 12 weeks (84 days) (*n* = 3 for each treatment).

Storage time	UDDT waste		UDDT waste + 0.5 % lime		UDDT waste + 2 % lime		UDDT waste + 5 % lime		U.S. biosolids	
	pH	MC (%)	pH	MC (%)	pH	MC (%)	pH	MC (%)	pH	MC (%)

Baseline	8.8	21	12.1	22	12.7	19	12.8	18	5.3	66
7 days	8.3	19	8.9	19	12.6	19	12.7	19	5.1	62
14 days	8.4	20	8.5	20	12.6	19	12.7	19	5.2	62
21 days	8.4	19	8.4	20	12.5	19	12.8	19	5.1	60
28 days	8.6	20	8.3	20	12.5	19	12.7	18	5.1	61
42 days	8.5	20	8.3	20	11.1	19	12.6	18	5.1	61
56 days	8.5	21	8.3	20	8.6	20	12.5	19	5.1	61
84 days	8.3	19	8.2	19	8.8	19	12.7	18	5.1	61

MC = moisture content.

**Table 3:** Mean log_10_ *E. coli* concentrations (CFU/g total solids) in untreated and lime-treated (0.5 %, 2 %, and 5 % (w/w)) UDDT waste and U.S. biosolids at 34 °C for 12 weeks (84 days).

	Mean Log_10_ *E. coli* CFU/g total solids (min–max)				
Storage time (days)	UDDT waste	UDDT waste + 0.5 % lime	UDDT waste + 2 % lime	UDDT waste + 5 % lime	U.S. biosolids

0	7.9 (7.8–8.0)^a^	1.2 (<0.7–2.2)	<0.7	<0.7	8.4 (8.2–8.6)
7	3.0	–	–	–	<1.3
	(2.8–3.3)				
14	2.0	–	–	–	<1.3
	(1.0–3.7)				
21	2.9	–	–	–	<1.3
	(2.1–3.4)				
28	3.1 (2.5–3.6)	–	–	–	<1.3
42^b^	–	–	–	–	<1.3
56	2.0	–	–	–	<1.3
	(<0.7–2.8)				
84	0.8	0.9 (0.7–1.1)	<0.7	<0.7	<1.3
	(<0.7–1.0)				

Note: No data were collected on day 7 to 56 for the lime-treated UDDT waste due to the large reductions observed at *t* = 0 and logistical constraints in the laboratory.

a Lower detection limits differ between UDDT waste and US biosolids due to moisture contents and back calculation to dry weight.

b Day 42 excluded from UDDT waste analysis due to error in sample measurement.

**Table 4:** Mean log_10_ reduction values (LRV) of Ascaris suum ova in control and lime-treated (0.5 %, 2 %, and 5 % (w/w)) UDDT waste and U.S. biosolids at 34 °C for 12 weeks (84 days).

Storage time	Mean LRV of viable *Ascaris suum* ova (no. of replicates with detectable viable ova)				
	UDDT waste	UDDT waste + 0.5 % lime	UDDT waste + 2 % lime	UDDT waste + 5 % lime	U.S. biosolids

7 days	0.4	0.2	2.4	≥2.5^d^	<0.1
	(3/3)	(3/3)	(2/3)	(0/3)	(3/3)
14 days	0.6	0.5	2.4	2.5	<0.1
	(3/3)	(3/3)	(1/3)	(1/3)	(3/3)
21 days	1.1	1.4	≥2.5	2.5	0.2
	(3/3)	(3/3)	(0/3)	(1/3)	(3/3)
28 days	2.4	2.5	2.5	≥2.5	0.1
	(2/3)	(2/3)	(1/3)	(0/3)	(3/3)
42 days^c^	–	–	–	–	0.4 (3/3)
56 days	≥2.5	≥2.5	≥2.5	≥2.5	1.7
	(0/3)	(0/3)	(0/2)	(0/3)	(3/3)
84 days	≥2.5	≥2.5	≥2.5	≥2.5	2.4
	(0/3)	(0/3)	(0/3)	(0/2)	(1/3)
T99^b^	21–28 days	21–28 days	0–7 days	0–7 days	56–84 days
p-value^a^	referent	0.5	0.008	0.001	0.01

a Paired sample *t*-test to individually compare mean LRVs of UDDT waste lime treatments and US biosolids to UDDT control at the day in which a ≥2-log reduction in either treatment was first observed.

b T_99_ = time to 99 % inactivation, or 2 log_10_ reduction.

c Results from day 42 are excluded in UDDT waste samples due to error in sample processing.

d The upper detection limit for log_10_ reduction values were capped at 2.5 log_10_ as a means to manage left-censored data; this capped detection limit reflects the largest volume observed after incubation (20 ml) and the lowest possible viable ovum count (1 ova).

## Data Availability

Data will be made available on request.

## HIGHLIGHTS

- Effective alternative sanitation options are needed in some humanitarian settings.
- UDDT performance was assessed in Hiloweyn Camp, Ethiopia.
- Substantial reductions in *E. coli* and *Ascaris* ova observed in stored UDDT waste.
- Hydrated lime addition may decrease required UDDT waste storage time.
- UDDTs could promote sustainable sanitation in comparable settings.

## Appendix A. Supplementary data

*Ascaris* viability methods; figures detailing *Ascaris* ova bag and indicator bag seeding in waste piles of UDDT vaults. Supplementary data to this article can be found online at [doi:https://doi.org/10.1016/j.scitotenv.2024.171838].

## Abbreviations

CDC: Centers for Disease Control and Prevention
CFU: Colony forming unit
EPA: Environmental Protection Agency
LRV: Log reduction value
MC: Moisture content
MPN: Most probable number
NRC: Norwegian refugee council
UDDT: Urine diverting dry toilet
UNHCR: United Nations High Commissioner for Refugees
WASH: Water, sanitation, and hygiene
WHO: World Health Organization
