# Supplementary material for: An environmental evaluation of urine-diverting dry toilets in Hiloweyn Camp, Dollo Ado, Ethiopia
Source: Sci Total Environ. Author manuscript; Available in PMC 2026 Apr 3. (PMC13047511; doi:10.1016/j.scitotenv.2024.171838)
Supplement: Supplementary Info_UDDT [file NIHMS1980246-supplement-Supplementary_Info_UDDT.docx]

**Supporting Information for:**

**An Environmental Evaluation of Urine-diverting Dry Toilets in Hiloweyn Camp, Dollo Ado, Ethiopia**

Travis W. Brown*^a,b^; Jennifer L. Murphy^b^; Patricia Akers^b^; Molly Patrick^a^; Vincent Hill^b^; Mia Mattioli^b^ Yegerem Tsige^c^.; Ahmed Adow^d^.; Mohamed Abdirashid^d^; Mohamed Nur Mohamed^d^; David Githiri^c^.; Thomas Handzel^a^

^a^ Emergency Response and Recovery Branch, Division of Global Health Protection, Centers for Disease Control and Prevention, Atlanta, GA, USA

^b^ Waterborne Disease Prevention Branch, Division of Foodborne, Waterborne, and Environmental Diseases, Centers for Disease Control and Prevention, Atlanta, GA, USA

^c^ United Nations High Commissioner for Refugees

^d^ Norwegian Refugee Council

*Address correspondence to Travis W. Brown(lue3@cdc.gov)

1600 Clifton Rd, Atlanta, GA 30329

Figures and Tables:
Figure S1 (page 4)
Figure S2 (page 5)

Table S1

**Materials & Methods**

*Ascaris viability*

*Ascaris* bags shipped to Atlanta were processed within 7 - 14 days of collection in Hiloweyn Camp and kept at 4°C until processing. *Ascaris* ova viability was determined using a filtration and flotation procedure followed by microscopy as previously described^1^. Briefly, sterile forceps were used to remove the *Ascaris* bag from each bottle, and sterile scissors were used to cut bags open. Contents were transferred into a sterile 50-mL conical tube by pouring followed by rinsing the bag with sterile DI water up to the 50 mL line of the tube to remove any residual waste. The tube was then vortexed for 30 seconds, allowed to settle for 30 minutes, and then vortexed for an additional 30 seconds. The contents were immediately filtered through a 300-micron sieve into a sterile 400-mL beaker, using 100 – 150 mL DI water to rinse both the sieve and conical tube. The filtered contents were transferred into the necessary number of 50-mL conical tubes and centrifuged at 800 *x g* for 10 minutes without brake. The resulting supernatant was removed by vacuum suction and 25 mL of a flotation solution (NaCl-saturated 500 g/L glucose solution) was added to each tube and the tube was vortexed for 30 seconds. The tubes were centrifuged at 800 *x g* for 10 minutes without brake and the supernatant was then filtered through a 38-micron sieve to capture ova. The top of the sieve was thoroughly rinsed with DI water into a sterile 150-mL beaker. The contents of the beaker were transferred into sterile 15-mL conical tubes, centrifuged at 800 *x g* for 10 minutes, and the resulting supernatant was vacuum-suctioned off. Approximately 10 mL of a 0.5% formalin solution was added to each tube, and tubes were centrifuged at 800 *x g* for 5 minutes. The resulting supernatant was removed down to approximately 0.5 mL. The pellet was resuspended and transferred into a glass Petri dish. Tubes were twice rinsed with approximately 0.5 mL of the formalin solution and poured into Petri dish. The Petri dish was sealed with paraffin film and incubated at 28 °C for a minimum of 28 days with gentle swirling every 2-3 days. A subset (0.25 – 0.50 mL) of the incubated solution was then transferred to a Sedgewick Rafter counting slide (Graticules Optics; Tonbridge, United Kingdom) and examined under 10X magnification to enumerate viable (i.e., containing larvae) and nonviable ova. *Ascaris* ova were considered viable if fully developed larvae were observed within ova (non-motile or motile)^2^. Counts were used to calculate viable *Ascaris* ova per gram of waste and log_10_ reduction values of ova (viable and nonviable) over time. Viable *Ascaris* ova log_10_ reductions are expressed as log_10_ (N_t_/N_0_), where N_0_ is the number of viable Ascaris ova at baseline and N_t_ is the number of viable Ascaris ova at time t.

**Figures**

**Figure S1.** Seeding location and depth of *Ascaris* ova bags and indicator bags within a UDDT waste pile: (A) view from the rear access doors with each oval representing one *Ascaris* ova bag and one indicator bag (B) view from above waste pile in relation to slab opening

**(A)**

**
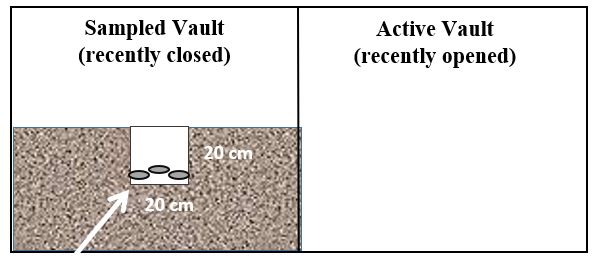
**

**(B)**

**
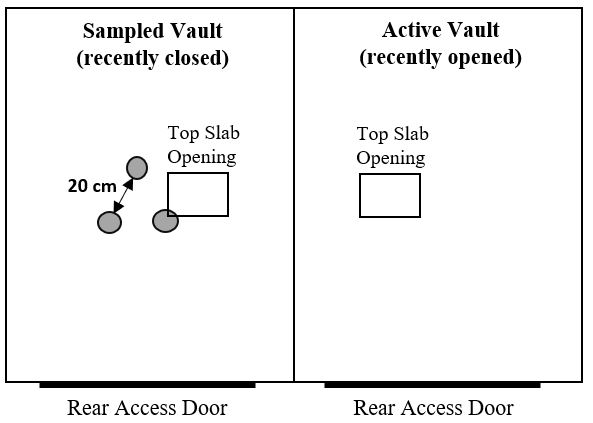
**

**Figure S2.** Diagram of sampling location within UDDT and transfer of bags and waste to 1 liter bottle prior to analysis.

**
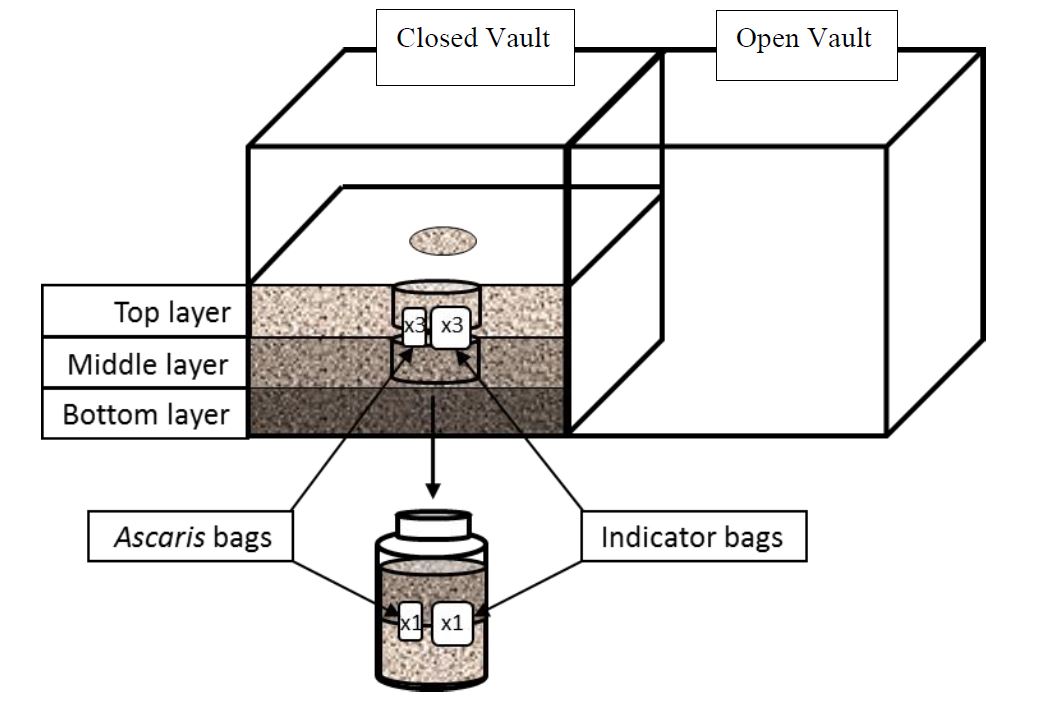
**

**Table S1.** Geometric mean *E. coli* concentrations in 20 shared-family UDDTs in Hiloweyn Camp not meeting the WHO *E. coli* guideline value (<1000 *E. coli* / g total solids) at baseline (0 months; September 2015) and after 6, 9, and 12 months of storage

| Storage Time | *E. coli* concentration (MPN / g total solids) | | |
| --- | --- | --- | --- |
|  | Mean | Min | Max |
| Baseline (n=14) | 6.3 x 10^7^ | 5.0 x 10^3^ | 1.2 x 10^8^ |
| 6 months (n=5) | 1.6 x 10^4^ | 4.0 x 10^3^ | 9.1 x 10^4^ |
| 9 months (n=2) | 6.1 x 10^3^ | 2.9 x 10^3^ | 1.3 x 10^4^ |
| 12 months (n=1) | >2.5 x 10^5^ | >2.5 x 10^5^ | >2.5 x 10^5^ |

**References**

(1) Roepstorff, A.; Nansen, P. Epidemiology, diagnosis and control of helminth parasites of swine. *FAO Animal Health Manual, Rome* **1998**. Vadlejch, J.; Petrtyl, M.; Zaichenko, I.; Cadkova, Z.; Jankovska, I.; Langrova, I.; Moravec, M. Which McMaster egg counting technique is the most reliable? *Parasitology research* **2011**, *109* (5), 1387-1394. DOI: 10.1007/s00436-011-2385-5 From NLM.

(2) Ravindran, V. B.; Shahsavari, E.; Soni, S. K.; Ball, A. S. Viability determination of Ascaris ova in raw wastewater: a comparative evaluation of culture-based, BacLight Live/Dead staining and PMA-qPCR methods. *Water Science and Technology* **2019**, *80* (5), 817-826. DOI: 10.2166/wst.2019.286 (acccessed 8/6/2021).
